# Knowledge and Clinical Practice Regarding Multiple Sclerosis Among Family Physicians in the First Health Cluster, Riyadh

**DOI:** 10.7759/cureus.102047

**Published:** 2026-01-22

**Authors:** Majed A Algaed, Omar H Alanazi, Saleh M Alzahrani, Saleh A AlKhaldi

**Affiliations:** 1 Family Medicine, First Health Cluster, Riyadh, SAU; 2 Research Center, King Fahad Medical City, Riyadh, SAU

**Keywords:** barriers, clinical practice, family physicians, knowledge, multiple sclerosis, primary care, referral, riyadh, saudi arabia

## Abstract

Background

The timely diagnosis of multiple sclerosis (MS) and patient referral in primary care are important. However, evidence on knowledge and clinical practice regarding MS among family physicians in Saudi Arabia (SA) is limited. This study aimed to assess knowledge and clinical practice regarding MS among family physicians in the First Health Cluster in Riyadh, SA.

Methodology

A cross-sectional study was conducted from May to December 2025. A total of 145 family physicians in hospitals and primary care centers have been recruited. Data were collected using a validated self-administered questionnaire on demographic characteristics and MS knowledge. Descriptive statistics and the chi-square test were used to explore associations between knowledge level and professional characteristics.

Results

Approximately 75 (51.7%) of participants showed high knowledge, and 64 (44.1%) had moderate knowledge. Common misconceptions were identified, particularly regarding the impact of MS on life expectancy 86 (59.3%) and the effects of pregnancy on MS 85 (58.6%). A total of 74 (51.0%) physicians reported seeing 1-5 patients with MS annually, whereas 65 (44.8%) had never encountered a patient with MS. Most physicians 86 (59.3%) preferred immediate referral. A low proportion of participants were confident in their ability to manage symptoms. Reported referral barriers included long waiting times (118; 81.4%), limited neurologist availability (58; 40.0%), and geographic distance (35; 24.1%).

Conclusions

There were significant gaps in prognosis and pregnancy-related knowledge, along with low confidence in symptom management and minimal initiation of work-up. These findings support targeted training, specifically for family medicine residency programs, the incorporation of MS-focused modules into residency curricula, and the development of national guidelines to strengthen MS care.

## Introduction

Multiple sclerosis (MS) is a chronic autoimmune demyelinating disease of the central nervous system and remains the most common cause of nontraumatic neurologic disability in young adults [1]. The usual age at onset is 20-40 years. Since 2020, approximately 2.8 million people live with MS worldwide, reflecting an increase in global prevalence [2]. Early diagnosis and intervention are important for improving outcomes, as prompt treatment can slow down disease progression and reduce long-term disability [3]. Despite advancements in disease-modifying therapies, MS care presents significant challenges in timely diagnosis, interdisciplinary management, and care standardization. MS is a complex condition that often requires coordination between primary and specialized care providers. Even minor delays or missteps in diagnosing early MS symptoms can lead to diagnostic delays, increased disease burden, and irreversible complications [4]. Persistent gaps in awareness and resources indicate that diagnostic delays occur even in well-resourced health systems, thereby underscoring the need for improved frontline diagnosis of MS [4].

Family physicians and other primary care providers play a pivotal role in MS care. This is because they are usually the first to encounter patients with symptoms that are indicative of MS and are essential for establishing a timely diagnosis and referral to neurologists. Considering the chronic nature of MS and the increasing clinical complexity of its management, many family physicians are involved in the long-term care of patients with MS [5]. With the growing burden of MS globally and in Saudi Arabia, the demand for knowledgeable primary care clinicians has increased. However, variations in knowledge, skills, and attitudes among primary care physicians remain a major barrier to effective MS care, potentially leading to misdiagnosis or delayed treatment initiation. Based on a recent international survey, 59% of countries lacked healthcare professional awareness about MS symptoms, which is a key barrier to early diagnosis [6]. In Saudi Arabia, where the prevalence of MS has increased to approximately 62 per 100,000 population, inconsistent familiarity with MS among general practitioners could exacerbate delays in patient care [7].

International studies have revealed that some physicians lack sufficient knowledge of and confidence in the management of MS, particularly in cases involving early or atypical presentations. The contributing factors include a lack of formal training programs, limited hands-on experience with MS cases, and the absence of unified care protocols in primary care. For example, a Saudi study conducted among family medicine residents, including 543 healthcare professionals across the Kingdom, found that only 13.8% had ever attended a conference or training on MS. Further, nearly half exhibited an overall poor knowledge of the disease [7]. Notably, knowledge gaps were most evident in recognizing MS symptoms, with that domain having the lowest scores, which can lead to primary care physicians overlooking the early signs of MS or misattributing them to benign conditions, thereby delaying referral and diagnosis [7].

Similarly, other studies have shown that inconsistent knowledge and attitudes among healthcare providers can significantly impact the quality and safety of MS care [8]. Al-Omar et al. revealed the need for national guidelines to streamline MS diagnosis and treatment and guide physicians in providing timely care. This emphasizes that improving standardization and education at the primary care level is essential for better patient outcomes [9].

The perceptions and attitudes of physicians toward managing chronic neurological diseases such as MS also influence their clinical behaviors and willingness to pursue ongoing professional development. A proactive attitude and structured, evidence-based training opportunities have been found to improve the confidence and competence of healthcare providers, leading to better patient outcomes. Various educational approaches, such as targeted workshops, case-based learning, and mentorship or collaborative care models, have improved the readiness of clinicians to handle MS cases. These include enhanced ability to identify relapses and manage routine care [10]. While primary care physicians manage a wide range of conditions and may face challenges in less prevalent diseases that require specialized hospital-based care, strengthening the competencies of primary care physicians via continuous medical education and the inclusion of MS management in the training curricula can facilitate the earlier diagnosis of MS and implement more effective co-management strategies with specialists. In turn, these measures are likely to translate into improved patient satisfaction, reduced emergency presentations, and overall safer care for people living with MS [10].

Despite an expanding body of global literature on MS, local studies examining the knowledge and clinical practices regarding MS among primary care physicians in Saudi Arabia are still limited. Only a few investigations have explored how family physicians in the region approach MS diagnosis, patient counseling, or adherence to contemporary guidelines. This gap makes it difficult for policymakers to design targeted training interventions or implement individualized, evidence-based guidelines based on local needs [11]. In the absence of such data, educational efforts may not completely address the specific misconceptions or weaknesses present in the primary care setting. Therefore, this study aimed to assess the knowledge and clinical practice regarding MS among family medicine physicians in the First Health Cluster, Riyadh. We believe that the outcomes of this research will be instrumental in identifying critical knowledge gaps and attitude patterns in this physician group.

## Materials and methods

A cross-sectional, descriptive-analytical study was conducted across primary care centers and institutions affiliated with First Health Cluster, Riyadh, from May to September 2025. The target population was family medicine physicians, which included PGY1-PGY3 residents, senior registrars, and consultants. The inclusion criteria were family medicine physicians currently practicing in the First Health Cluster, Riyadh. The exclusion criteria were limited to physicians with incomplete questionnaire responses.

Sample size and sampling technique

The sampling frame included all eligible family physicians working in primary care centers within the chosen geographic area. To determine the minimum required sample size, the finite population correction formula was used, given that the total population of eligible participants was 223: n = (N × Z² × P (1 - P)) / (d² × (N - 1) + Z² × P (1 - P)), where N = 223 (total population of eligible family physicians), Z = 1.96 (corresponding to a 95% confidence level), P = 0.5 (expected proportion; used when no prior estimate is available), and d = 0.05 (margin of error). Substituting values: n = (223 × 1.96² × 0.5 × (1 - 0.5)) / (0.05² × (223 - 1) + 1.96² × 0.5 × (1 - 0.5)) = 142.

Data collection tool

Data were collected using a structured, self-administered questionnaire developed by the research team. We followed the validation and verification process, where two experts supervised and reviewed the content. Subsequently, a pilot study was conducted to confirm clarity and reliability.

Tool Structure and Scoring

Section A: Demographic and professional characteristics (such as age, sex, workplace/center, professional level, and years of experience).

Section B: Knowledge of MS: 10 multiple-choice items covering core domains (e.g., pathophysiology/autoimmunity, age and sex patterns, prognosis, pregnancy, sentinel presentations such as optic neuritis diagnosed based on MRI results, and the role of disease-modifying therapies). Each correct answer was assigned 1 point, and incorrect or “don’t know” responses received 0 points, resulting in a total score of 0-10. Knowledge levels were categorized as: High: ≥8, Moderate: 5-7, and Low: <5. These cut-off points were adapted from scoring approaches commonly used in similar MS knowledge assessments in prior studies.

Section C: Clinical practice: items capturing annual MS caseload, frequency of initiating investigations for suspected MS, and comfort management for common MS-related symptoms (such as fatigue, spasticity, and bladder dysfunction).

Section D: Referral practices: timing of referral after initial suspicion and a checklist of perceived barriers to specialist referral (e.g., limited neurologist availability, long waiting times, geographic distance, and patient reluctance).

Data collection procedure

After site coordination with facility leads, electronic invitations were sent to eligible physicians, accompanied by an information sheet that provided details on the purpose of the study, voluntary participation, confidentiality assurances, and consent statement. The participants completed the questionnaire at their own convenience within the data collection window.

Variables and operational definitions

Primary outcome (knowledge level): total score in Section B (0-10) and its categorical classification (high/moderate/low) as defined above. Practice indicators: self-reported annual MS caseload (none, 1-5, 6-15, and >15), frequency of initiating investigations (frequently/occasionally/rarely/never), and confidence in symptom management (very/moderately/slightly/not confident). Referral indicators: timing of referral (immediate vs. after the initial investigations/confirmation) and perceived barriers (multiple choices).

Statistical analysis

Analyses were conducted using the SPSS software version 25 (IBM Corp., Armonk, NY, USA). Descriptive statistics (frequencies and percentages) were used to summarize all categorical variables, including characteristics of the participants, item-level knowledge responses, and clinical practice/referral patterns. Associations between categorical variables (knowledge level vs. professional level and years of experience) were examined using the chi-square test. A p-value <0.05 was considered statistically significant.

Data management plan

Data were collected by the investigators using secure electronic forms. All records were de-identified at entry and assigned unique study codes. No identifying information was retained in the analytic dataset. Data were stored on the principal investigator’s password-protected device, with encrypted backup on an institutionally approved drive. Access was restricted to authorized members of the team. Quality checks (e.g., range checks and logic rules) were performed before analysis.

Ethical considerations

The study obtained ethical approval from the Institutional Review Board at the Ministry of Health, Saudi Arabia (approval number: H1Q1-10-Apr25-02). Furthermore, it was conducted in accordance with the tenets of the Declaration of Helsinki and adhered to the Guidelines of Good Clinical Practice. Electronic informed consent was obtained before accessing the questionnaire. Participants were informed about the study objectives, procedures, and potential benefits, and they had the right to withdraw at any time without penalty. Confidentiality of the data was assured, and the privacy of the participants was protected.

## Results

In total, 145 family physicians participated in this study. As shown in Table 1, the majority of participants were aged 26-30 years (94, 64.8%), representing more than half of the sample. Meanwhile, physicians aged 20-25 years (7, 4.8%) constituted the smallest age group. Regarding sex distribution, male physicians accounted for 79 (54.5%), slightly higher than female physicians at 66 (45.5%). The participants were affiliated with various centers. King Saud Medical City (KSMC) had the largest proportion (93, 64.1%), followed by Prince Sultan Health Center (17, 11.7%). Smaller proportions were distributed across Shifa1-PHC (10, 6.9%), Alzahra Center (6, 4.1%), Alshimisy (3, 2.1%), Riyadh First Health Cluster (3, 2.1%), and other centers (13, 9.0%). Regarding professional level, most respondents were at the PGY1 level (59, 40.7%), while senior registrars and consultants together accounted for 30 (20.7%). In terms of work experience, more than half of the physicians (75, 51.7%) had ≤2 years of experience, indicating a predominantly early-career population, and only 3 (2.1%) had more than 16 years of experience (Table 1).

**Table 1:** Demographic and professional characteristics of the participants (N = 145).

Variable	Category	N (%)
Age (years)	20–25	7 (4.8%)
	26–30	94 (64.8%)
	31–35	29 (20.0%)
	>35	15 (10.3%)
Sex	Male	79 (54.5%)
	Female	66 (45.5%)
Hospital/Center name	KSMC	93 (64.1%)
	Shifa1-PHC	10 (6.9%)
	Riyadh First Health Cluster	3 (2.1%)
	Prince Sultan Health Center	17 (11.7%)
	Alzahra Center	6 (4.1%)
	Alshimisy	3 (2.1%)
	Others	13 (9.0%)
Professional level	PGY1	59 (40.7%)
	PGY2	31 (21.4%)
	PGY3	25 (17.2%)
	Senior Registrar	23 (15.9%)
	Consultant	7 (4.8%)
Experience level	≤2 years	75 (51.7%)
	3–5 years	50 (34.5%)
	6–10 years	13 (9.0%)
	11–15 years	4 (2.8%)
	16+ years	3 (2.1%)

Several knowledge items demonstrated high correct response rates in this study, ranging from 85.5% to 93.1%, including recognition of MS as a disease of the immune system (133, 91.7%), identification of the role of disease-modifying therapies in preventing disease progression and reducing relapses (135, 93.1%), selection of MRI as the most useful diagnostic tool (126, 86.9%), and awareness that MS affects women more commonly than men (124, 85.5%). In contrast, lower correct response rates were observed for recognizing that MS does not significantly shorten lifespan (59, 40.7%) and reporting that pregnancy does not worsen MS (60, 41.4%). See Table 2 for more details.

**Table 2:** Assessment of knowledge regarding multiple sclerosis among family physicians (N = 145). The table shows the distribution of correct and incorrect responses provided by family physicians regarding their knowledge of multiple sclerosis (MS).

Knowledge statement	Incorrect responses (%)	Correct responses (%)
MS is a disease of	34 (23.4%)	111 (76.6%)
MS significantly shortens the lifespan	86 (59.3%)	59 (40.7%)
MS is a disease of the immune system	12 (8.3%)	133 (91.7%)
MS typically occurs at age	35 (24.1%)	110 (75.9%)
MS occurs in	21 (14.5%)	124 (85.5%)
Pregnancy worsens MS	85 (58.6%)	60 (41.4%)
There is no cure for MS	41 (28.3%)	104 (71.7%)
Most common initial symptom	54 (37.2%)	91 (62.8%)
Diagnostic test for MS	19 (13.1%)	126 (86.9%)
Role of disease-modifying therapies in MS	10 (6.9%)	135 (93.1%)

Based on their responses to the 10-item knowledge assessment, 75 (51.7%) physicians had a high level of knowledge, accurately answering at least 80% of the questions, while 64 (44.1%) were classified as having moderate knowledge with scores ranging from 50% to 79%. Notably, 6 (4.1%) participants fell below the 50% threshold, indicating a low level of knowledge.

The majority of the participants (37, 56.9%) reported seeing one to five patients with MS annually, whereas 25 (38.5%) indicated that they did not encounter patients with MS in their clinical practice. Regarding diagnostic behavior, only 2 (3.1%) physicians frequently initiated investigations for suspected MS, while most either rarely (21, 32.3%) or never (29, 44.6%) started investigations themselves, opting instead to refer patients directly to specialists (Table 3). When asked about their confidence level in managing MS-related symptoms such as fatigue, spasticity, and bladder dysfunction, nearly half of the physicians (29, 44.6%) stated that they were not confident at all, while only 4 (6.2%) felt very confident. In terms of referral timing, most participants (40, 61.5%) referred patients to a neurologist immediately upon suspicion of MS, while others delayed referrals until after initial investigations (17, 26.2%) or confirmation through specialized tests (4, 6.2%). See Table 3 for more details.

**Table 3:** Clinical practice patterns and referral behaviors among family physicians (N = 145). The table shows the clinical practices and referral behaviors of family physicians in managing multiple sclerosis (MS) cases.

Item	Category	N (%)
Patients with MS seen annually	None	65 (44.8%)
	1–5	74 (51.0%)
	6–15	6 (4.1%)
Initiate investigations for suspected MS	Frequently	9 (6.2%)
	Occasionally	20 (13.8%)
	Rarely	37 (25.5%)
	Never (always refer)	79 (54.5%)
Comfort in managing MS symptoms	Very comfortable	12 (8.3%)
	Moderately comfortable	30 (20.7%)
	Slightly comfortable	41 (28.3%)
	Not comfortable at all	62 (42.8%)
Referral timing after MS suspicion	Immediately upon suspicion	86 (59.3%)
	After initial investigations	35 (24.1%)
	After confirmation via tests	11 (7.6%)
	Rarely or never	13 (9.0%)

**Figure 1:**
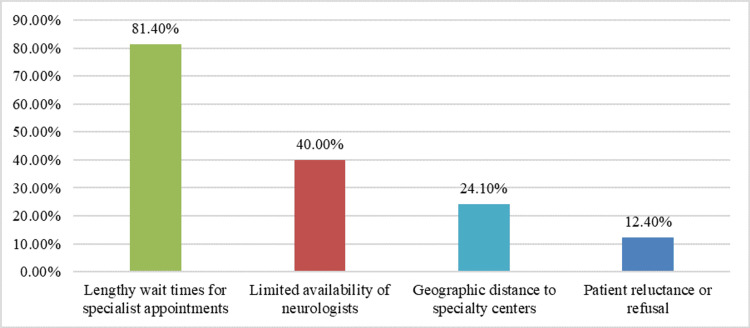
Barriers to multiple sclerosis (MS) specialist referral. The figure demonstrates the key barriers faced by family physicians when referring patients with suspected or confirmed MS to specialists.

The most commonly reported obstacle was the lengthy wait time for specialist appointments, as cited by 118 (81.4%) participants. In addition, 58 (40.0%) physicians reported limited neurologist availability as a referral barrier. Geographic distance to specialty centers was also reported by 35 (24.1%) respondents. Notably, 18 (12.4%) participants reported patient reluctance or refusal as a barrier (Figure 1).

The distribution of knowledge levels across professional ranks showed that participants from all levels had a mix of high and moderate knowledge. PGY1 physicians accounted for the largest proportion of the sample (59, 40.7%), including 26 (17.9%) with high knowledge and 29 (20%) with moderate knowledge. Senior registrars and PGY3 physicians demonstrated relatively balanced distributions between knowledge levels. However, no statistically significant association was found between professional level and knowledge level. Regarding years of clinical experience, physicians with 0-2 years (33, 22.8%) and 3-5 years (30, 20.7%) comprised nearly half of the sample (n = 63, 44% combined) and exhibited higher knowledge levels compared with other groups (Table 4).

**Table 4:** Professional characteristics and overall knowledge level (N = 145). The table presents the association between professional characteristics and the overall knowledge level on multiple sclerosis (MS) among family physicians.

Variables	Category	High knowledge (%)	Moderate knowledge (%)	Low knowledge (%)	Total (%)	P-value
Professional level	PGY1	26 (17.9)	29 (20.0)	4 (2.8)	59 (40.7)	0.673
	PGY2	18 (12.4)	12 (8.3)	1 (0.7)	31 (21.4)	
	PGY3	15 (10.3)	9 (6.2)	1 (0.7)	25 (17.2)	
	Senior registrar	11 (7.6)	12 (8.3)	0 (0.0)	23 (15.9)	
	Consultant	5 (3.4)	2 (1.4)	0 (0.0)	7 (4.8)	
Experience level	≤2 years	33 (22.8)	38 (26.2)	4 (2.8)	75 (51.7)	0.412
	3–5 years	30 (20.7)	18 (12.4)	2 (1.4)	50 (34.5)	
	6–10 years	7 (4.8)	6 (4.1)	0 (0.0)	13 (9.0)	
	11–15 years	4 (2.8)	0 (0.0)	0 (0.0)	4 (2.8)	
	16+ years	1 (0.7)	2 (1.4)	0 (0.0)	3 (2.1)	

## Discussion

This study aimed to assess the knowledge and clinical practice regarding MS among family physicians in the First Health Cluster, Riyadh. Findings revealed that although 75 (51.7%) participants demonstrated high levels of knowledge, notable gaps persisted in specific clinical aspects. Most physicians correctly identified MS as a disease of the central nervous system and recognized its autoimmune nature; however, substantial misconceptions were observed regarding the impact of pregnancy on MS and the effect of the disease on life expectancy. Furthermore, while 74 (51.0%) respondents reported encountering one to five MS patients annually, a considerable proportion (65, 44.8%) had never encountered any. Most physicians preferred to refer suspected MS cases immediately to neurologists rather than initiating diagnostic investigations or managing symptoms independently. Statistical analysis revealed no significant association between knowledge level and either professional rank (p = 0.673) or years of experience (p = 0.412).

These findings are consistent with previous regional research showing moderate-to-good knowledge levels among primary care physicians, coupled with persistent deficiencies in specialized domains. For instance, Halabi et al. reported that 56.9% of family medicine residents in Makkah demonstrated good knowledge, with particularly low recognition of early symptoms such as optic neuritis, similar to the present findings, where 91 (62.8%) respondents identified optic neuritis as the most common initial manifestation [12]. Comparable results were also reported by Alfarawi et al. in Riyadh, who found adequate general awareness among physicians but noted knowledge gaps in complex clinical features such as cognitive decline and Lhermitte’s phenomenon [13]. Collectively, these data indicate that although physicians are familiar with the basic aspects of MS, they often lack deeper clinical insight.

The current results also highlight the limited clinical engagement of family physicians in MS management. Only 9 (6.2%) participants frequently initiated investigations for suspected MS, while most (116, 80.0%) reported rarely or never doing so. This conservative approach is consistent with the findings of Al-Omar et al., who noted that neurologists in Saudi Arabia often discourage general practitioners from initiating workups due to concerns regarding expertise [9]. Such hesitancy may contribute to diagnostic delays and fragmented referral pathways, as echoed by international evidence emphasizing that general practitioners are usually the first point of contact but remain underutilized in early MS detection [8]. The absence of a statistically significant relationship between knowledge and years of experience (p = 0.412) or professional level (p = 0.673) in this study does not allow for definitive conclusions regarding whether newer physicians possess more updated theoretical knowledge or lack practical competence. However, this finding may suggest that factors other than years of experience or professional seniority, such as training quality and institutional support, could play a more significant role in knowledge variation. Conversely, Halabi et al. found that PGY4 residents demonstrated significantly higher knowledge levels (83.3%) compared to PGY2 and PGY3 residents (47.2% and 47.4%, respectively), indicating that training quality and institutional support may influence knowledge variation more than seniority alone [14].

A notable aspect of this study is the limited comfort in managing MS-related symptoms, reported by 62 (42.8%) participants. This mirrors global findings such as those by Mackowiak et al., who demonstrated that only a small proportion of general practitioners could accurately define MS relapse despite believing they could manage it clinically [15]. The discrepancy between perceived ability and actual competence underscores the importance of structured continuing medical education. Moreover, the observation that 44.8% of physicians do not encounter MS patients yearly, despite registry data suggesting most patients initially present in primary care, suggests possible under-recognition or misclassification of MS at early stages, a concern also noted in national reports [7].

The pattern of misconceptions and limited comfort identified among Saudi family physicians aligns with broader community-level studies showing insufficient MS awareness. Several population-based studies across Saudi Arabia, including those conducted in Al-Ahsa [16], Jeddah [17], and Qassim [18], have reported suboptimal understanding of MS symptoms, risk factors, and prognosis. Similarly, Dahlawi et al. found that most individuals in the Western Region had limited knowledge about MS [19], while an older study in Riyadh demonstrated comparable gaps [20]. These findings indicate that both healthcare professionals and the general public share insufficient awareness, emphasizing the urgent need for comprehensive national education strategies.

Regarding referral barriers, Figure 1 shows that lengthy waiting times for specialist appointments (118, 81.4%) and limited neurologist availability (58, 40.0%) emerged as the most common challenges. Geographic distance (35, 24.1%) and patient reluctance (18, 12.4%) were also cited. These barriers parallel those documented in international studies, including Pétrin et al., who noted that delays and poor communication between primary and specialist care compromise timely diagnosis [21]. Addressing these structural obstacles requires policy-level interventions aimed at improving neurologist accessibility and optimizing integrated care models. Regional initiatives should also focus on strengthening communication between general practitioners and MS centers to facilitate early referral and follow-up. Recent regional studies among MS patients have also revealed insufficient disease knowledge among patients themselves, suggesting a broader educational gap that extends beyond healthcare providers. For instance, a study found that most Saudi MS patients had a limited understanding of their condition [22]. Moreover, another study highlighted the increasing prevalence of MS across Gulf countries and the pressing need for interprofessional awareness to reduce diagnostic delays [23].

This study has a number of limitations. Although it is a multi-center study, its population may not represent the majority, as the study only covers part of the central region of the country, which might have limited the generalizability of the findings. In addition, as the data were collected using structured questionnaires rather than direct observations, some aspects of clinical practice might not have been completely captured, which could affect the depth of assessment regarding actual performance.

## Conclusions

In conclusion, this study contributes to a growing body of regional and global literature showing that family physicians generally possess foundational knowledge on MS. However, significant deficiencies remain in less prominent clinical aspects and management strategies. These findings support previous calls by researchers and policy makers for targeted education programs, practical clinical training, and the development of national MS care guidelines tailored to the Saudi context. Empowering family physicians with the necessary diagnostic and management skills is important for reducing delays in specialist referral and enhancing patient outcomes in a healthcare system increasingly burdened by chronic neurological diseases such as MS. Furthermore, a future study with bigger sample size, larger geographical area and focus groups is highly recommended.

## Appendices

**Table 5:** Data collection tool.

Age	☐ 20–25 ☐ 26–30 ☐ 31–35 ☐ >35
Gender	☐ Male ☐ Female
Do you work at Riyadh First Health Cluster?	☐ Yes ☐ No
If yes, name of Hospital/Center:	___________________________________
Level	☐ PGY1 ☐ PGY2 ☐ PGY3 ☐ Senior Registrar ☐ Consultant
Work Experience (years):	_________________________

Multiple sclerosis is a disease of:	☐ The central nervous system ☐ All body organs ☐ Don’t know
Multiple sclerosis significantly shortens lifespan:	☐ True ☐ False ☐ Don’t know
Multiple sclerosis is a disease of the immune system:	☐ True ☐ False ☐ Don’t know
Multiple sclerosis can manifest at any age, but typically occurs:	☐ Before 20 years ☐ Between 20–40 years ☐ Between 40–60 years ☐ Don’t know
Multiple sclerosis occurs in:	☐ Women and men about equally ☐ Men about twice as often as women ☐ Women about twice as often as men ☐ Don’t know
Pregnancy worsens multiple sclerosis:	☐ True ☐ False ☐ Don’t know
At present, there is no treatment that can cure multiple sclerosis:	☐ True ☐ False ☐ Don’t know
Most common initial presenting symptom of MS:	☐ Seizures ☐ Fatigue ☐ Optic neuritis ☐ Tremor
Diagnostic test most helpful in confirming MS:	☐ CT scan of the brain ☐ Nerve conduction study ☐ MRI of the brain and spinal cord ☐ Don’t know
Role of disease-modifying therapies (DMTs):	☐ Cure the disease ☐ Alleviate acute symptoms ☐ Prevent disease progression and reduce relapses ☐ Treat spasticity only

Approximately how many MS patients do you see annually?	☐ None ☐ 1–5 ☐ 6–15 ☐ More than 15
How often do you initiate investigations for suspected MS?	☐ Frequently ☐ Occasionally ☐ Rarely ☐ Never (always refer)
How confident are you in managing symptoms related to multiple sclerosis (e.g., spasticity, fatigue, bladder dysfunction)?	☐ Very confident ☐ Moderately confident ☐ Slightly confident ☐ Not confident at all

How soon after initial suspicion of MS do you typically refer patients to a neurologist?	☐ Immediately upon suspicion ☐ After initial investigations ☐ After confirmation by further specialized tests ☐ Rarely or never
What barriers do you face when referring patients with suspected or confirmed MS to specialists? (Check all that apply)	☐ Limited availability of neurologists ☐ Lengthy wait times for specialist appointments ☐ Geographic distance to specialty centers ☐ Patient reluctance or refusal
